# Quantifying heterogeneity in an animal model of acute respiratory distress syndrome, a comparison of inspired sinewave technique to computed tomography

**DOI:** 10.1038/s41598-024-55144-z

**Published:** 2024-02-28

**Authors:** Minh C. Tran, Douglas C. Crockett, Tu K. Tran, Phi A. Phan, Formenti Federico, Richard Bruce, Gaetano Perchiazzi, Stephen J. Payne, Andrew D. Farmery

**Affiliations:** 1 Nuffield Division of Anesthetics, Nuffield Department of Clinical Neurosciences, University of Oxford, Level 6, West Wing, John Radcliffe Hospital, Oxford, OX3 9DU UK; 2 Department of Engineering and Science, University of Oxford, Oxford, UK; 3 Centre for Human and Applied Physiology, King’s College London, London, UK; 4 Hedenstierna Laboratory, Department of Surgical Sciences, Uppsala University, Uppsala, Sweden; 5 Department of Biomechanics, The University of Nebraska Omaha, Omaha, USA

**Keywords:** Lung heterogeneity, Inspired sinewave test, Computed tomography, PEEP, Respiration, Respiratory distress syndrome, Biomedical engineering

## Abstract

The inspired sinewave technique (IST) is a non-invasive method to measure lung heterogeneity indices (including both uneven ventilation and perfusion or heterogeneity), which reveal multiple conditions of the lung and lung injury. To evaluate the reproducibility and predicted clinical outcomes of IST heterogeneity values, a comparison with a quantitative lung computed tomography (CT) scan is performed. Six anaesthetised pigs were studied after surfactant depletion by saline-lavage. Paired measurements of lung heterogeneity were then taken with both the IST and CT. Lung heterogeneity measured by the IST was calculated by (a) the ratio of tracer gas outputs measured at oscillation periods of 180 s and 60 s, and (b) by the standard deviation of the modelled log-normal distribution of ventilations and perfusions in the simulation lung. In the CT images, lungs were manually segmented and divided into different regions according to voxel density. A quantitative CT method to calculate the heterogeneity (the Cressoni method) was applied. The IST and CT show good Pearson correlation coefficients in lung heterogeneity measurements (ventilation: 0.71, and perfusion, 0.60, *p* < 0.001). Within individual animals, the coefficients of determination average ventilation (R^2^ = 0.53) and perfusion (R^2^ = 0.68) heterogeneity. Strong concordance rates of 98% in ventilation and 89% when the heterogeneity changes were reported in pairs measured by CT scanning and IST methods. This quantitative method to identify heterogeneity has the potential to replicate CT lung heterogeneity, and to aid individualised care in ARDS.

## Introduction

The recent coronavirus pandemic caused > 6.5 million recognized deaths worldwide and highlighted the impact that severe acute respiratory distress syndrome (ARDS) has on healthcare systems^1^. Mechanical ventilation is a life-saving therapy required by nearly half of ARDS patients. However, with the exception of some basic ground rules, there is no clear guidance for nuanced ventilatory management in ARDS^2,3^. Ventilator induced lung injury is thought to occur mainly through two mechanisms: *atelectotrauma*, the opening/collapsing of lung units, and *volutrauma*, the repetitive over-inflation of the lung^4–6^. To prevent ventilator-induced lung injury, endpoints by which Positive End-Expiratory Pressure (PEEP) might be titrated for individual patients remains elusive^7,8^. Lung heterogeneity may provide an individualised ventilatory target.

Ventilatory heterogeneity is the “uneven ventilation” or the unequal distribution of the airflow (ventilation) relative to the size of the lung regions to which it is distributed^9,10^. Ventilation heterogeneity and perfusion heterogeneity of the lung have been considered as key research points of lung diseases^11–13^. Computed tomography (CT) measured lung heterogeneity increases with severity of disease in ARDS^14,15^. Quantification of ventilatory heterogeneity has the potential to benefit patients with lung disease^16–18^. CT scanning is widely considered the gold-standard to determine lung recruitability, effective lung volume, and ventilatory heterogeneity to help determine preliminary PEEP selection^19^. Work by Cressoni, et al. in 148 ARDS patients showed the potential of quantitative CT image analysis to determine lung inhomogeneity. However, CT scanning is labour intensive and not without risks.

The Inspired Sinewave Technique (IST) was developed to measure deadspace volume, effective lung volume, pulmonary blood flow, and lung heterogeneity continuously and non-invasively at the bedside, without the need for patient co-operation, with an operator-independent approach, and not requiring ionising radiation^20^. The IST adds a forced oscillation of tracer gas into inspiratory airflow to measure cardiopulmonary parameters^20,21^. Additionally, the IST in conjunction with advanced lung simulation can determine ventilation and perfusion heterogeneity within the lung^22^. Given the limitations and risks of CT scanning, a simple and non-invasive alternative technology, like the IST, has a clear clinical need. Preliminary results of this work have been published previously^23^ wherein simple CT quantitative analysis, using the ratio between atelectatic and over-extended lung, was used to identify heterogeneity of the lung, and compared to IST measurements. However, this is the first study comparing Cressoni’s CT analysis with IST heterogeneity, and comparing IST with simulation-based calculations of the lung heterogeneity^24^.

We hypothesised that lung heterogeneity values measured by the CT imaging could be similar to the bedside IST values in the ARDS porcine model. We then further investigated the changes in lung heterogeneity with the changes in different PEEP levels to suggest that measuring lung heterogeneity at the bedside has the potential to optimise the ventilator setting to provide better support for ARDS subjects.

## Methods

### Ethical approval

This study was approved by Animal Research Ethics Committee at Uppsala University (Ref: C98/16). All the animal experiments conformed to the National Institutes of Health Guidelines for the Use of Laboratory Animals. We abide by the Animal Research: Reporting of In Vivo Experiments guidelines (ARRIVE)^25^. Previous ethical documents were included here^26^. All methods were performed following the relevant guidelines and regulations by ARRIVE, APPROVAL, and ACCORDANCE.

### Protocol

Six anaesthetised pigs underwent surfactant depletion by saline lavage. The summary baseline data are shown in Table 1. The animals were premedicated with i.m. xylazine 2 mg/kg, ketamine 20 mg/kg, and midazolam 0.5 mg/kg^27^. Then, animals underwent induction of anaesthesia with i.v. propofol titrated to effect (1–3 mg/kg). The trachea was intubated. Volume-control ventilation was implemented with Servo-I, Maquet ventilator (Rastatt, Germany) at 20–25 breaths per minute, and with a tidal volume of 10 mL/kg. The inspiration:expiration ratio was 1:2. Surfactant depletion was undertaken according to the Lachmann method^28^. This process was repeated until the PaO_2_:F_I_O_2_ ratio (PFR) was < 300 mmHg at a PEEP level of 5 cmH_2_O and F_I_O_2_ 0.7.

**Table 1:** Baseline characteristics of n = 6 pigs.

Parameter	Animal number						Mean	SD
	1	2	3	4	5	6		
Weight (kg)	31	32	28	28	31	32	30	1.6
HR	134	80	87	81	95	120	100	20.4
FiO_2_	90	77	88	75	89	88	84.5	6.1
PaO_2_ (kPa)	28.6	26.4	15.6	23.9	10.3	20.6	20.9	6.3
PaCO_2_ (kPa)	8.0	7.1	6.3	6.7	7.1	7.9	7.2	0.6
P/F R (mmHg)	268	248	156	239	87	176	196	63
pH	7.24	7.35	7.36	7.34	7.29	7.18	7.30	0.07
CO (L/min)	4.0	3.2	3.2	2.9	3.7	3.1	3.4	0.4
SaO_2_ (%)	98.1	98.3	97.0	99.9	98.3	98.2	98.3	0.8

Blood gas data were measured after the saline lavage. FiO_2_ is fraction of inspired O_2_; CO is cardiac output; P/F R is PaO_2_:FiO_2_ ratio; SaO_2_ is saturation of O_2_; SD is standard deviation.

During the experiments, the PEEP level was incrementally increased from 5 through to 10, 15 and 20 cmH_2_O and decreased back to 15, 10 and 5 cmH_2_O. At each PEEP level, two paired measurements of lung volume by the IST and CT were performed. Supplementary Figure A1 shows a summary timeline of each measurement.

### Lung heterogeneity measured by the IST

The IST uses a forced oscillation of a low-dose tracer gas (N_2_O) in the inspired air with a set sinusoidal period^20,22,26,29,30^. Measurements using IST were made using sinusoid periods of both 60 s and 180 s. By measuring the system output (in the expired gas), compared to the forced input signal, and applying mass conservation principles, the IST analysis allows the calculation of the lung parameters. Recoverable variables of IST are deadspace volume (V_D_), (volume not involved in gas exchange); effective lung volume (ELV), (the apparent volume of the alveolar compartment, assuming it to be heterogeneously ventilated); pulmonary blood flow (Qp), and lung heterogeneity index (H), which quantifies the heterogeneity of the lung, as both ventilation and perfusion per unit lung compartment volume.

**Figure 1:**
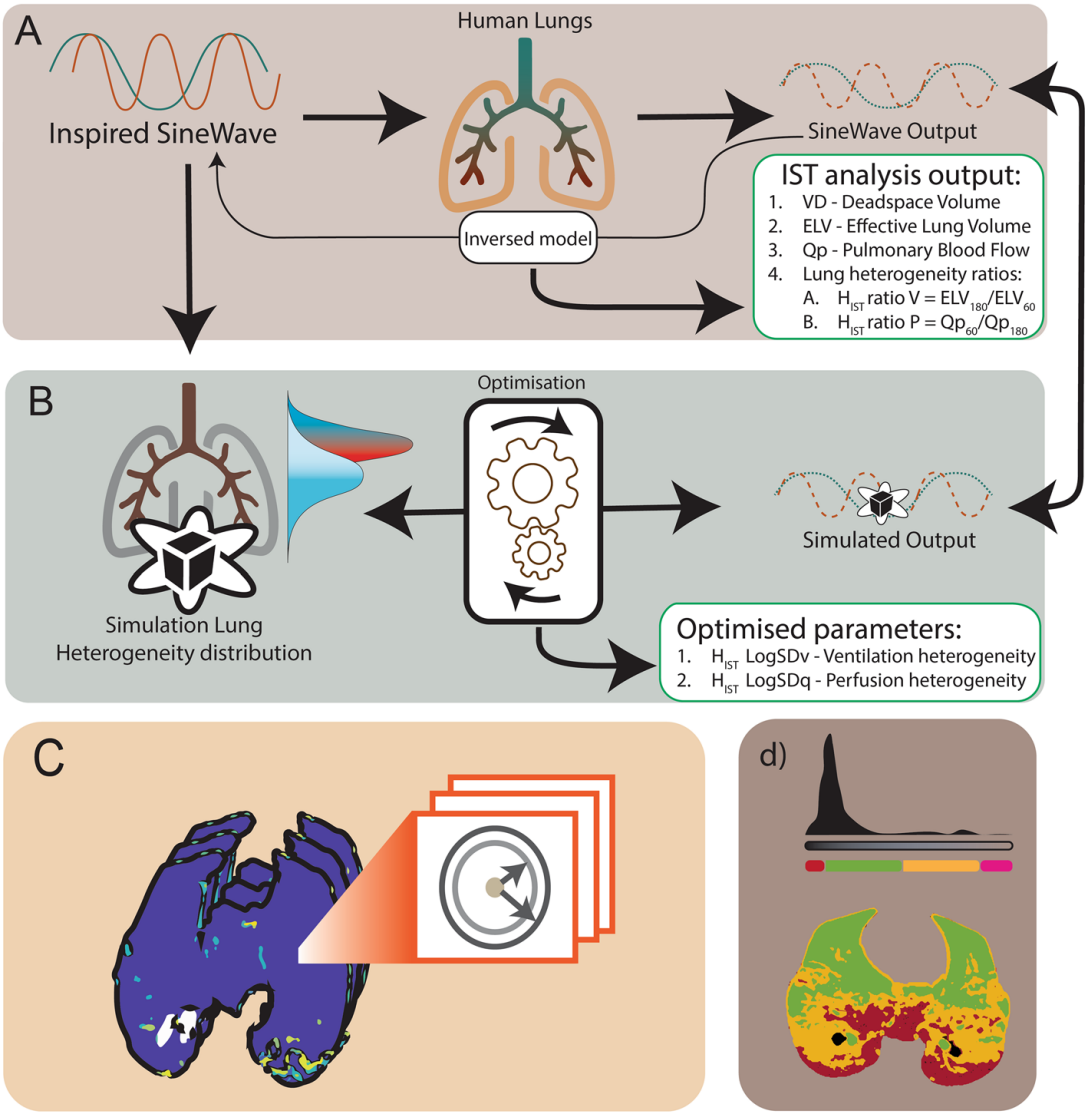
Methods to measure lung heterogeneity by IST and CT images. Panel (**A**) shows the working principle of the IST, using N_2_O tracer-gas and applied an inversed model to calculate pulmonary-vascular parameters. Panel (**B**) shows a schematic diagram of a method to optimise the heterogeneity simulation results versus measured IST results to calculate the ventilation/perfusion heterogeneity. Panels (**C**) and (**D**) illustrate two quantitative methods to calculate the heterogeneity index from CT imaging scans. Panel (**C**) shows compare surrounding voxel method (Cressoni’s method) and panel (**D**) shows the classification method based on Hounsfield unit ranges.

In this study, we focus upon two methods to measure heterogeneity by the IST. A schematic diagram of both calculations is presented in Fig. 1A and B. The conventional IST method exploits the fact that errors in the ELV estimated by the IST are increasingly frequency-dependent as heterogeneity increases. The ratio of the ELV outputs at 60 s and 180 s oscillation periods will therefore deviate from 1.0 as heterogeneity increases, in Fig. 1A. Further details of this method are discussed here^30^. In this method the IST heterogeneity ratios (${H}_{IST} ratios$) are calculated by the ratios of the ELV_180_/ELV_60_ for ventilation, and Qp_60_/Qp_180_ for perfusion.

In the second method, the IST heterogeneity indices are calculated using a multi-compartment simulation model, in Fig. 1B. The same oscillatory signals that are fed into the animal are input into the simulation which is a multi-compartment model with log-normally distributed specific ventilations and perfusions, to represent the real lung. The optimisation process adjusts this simulation such that its output best matches the output from the experimental animal. The simulation-based optimisation therefore recovers the log-normal distribution of specific ventilation and perfusion of the lung. Then, the width of these distributions within the simulation (the log standard deviations) will now represent lung heterogeneity. LogSDv presents the ventilatory heterogeneity and LogSDp is the perfusion heterogeneity (called the H_IST_ LogSD indices). Further details and mathematical equations of the optimisation process are presented in^24^, which could be summarised in the optimisation of the loss function:

$$ L\left( {LogSD} \right) = \left| {y_{180s} - \hat{y}\left( {LogSD} \right)} \right| + \left| {y_{60s} - \hat{y}\left( {LogSD} \right)} \right| $$

where $$y_{180s}$$ is the IST results measurement at 180 s, similar to the $$y_{60s}$$, $$\hat{y}\left( {LogSD} \right)$$ is the lung simulation controlled by the $$LogSD $$ in both perfusion and ventilation.

### Measurement of the lung heterogeneity using CT scan

A Somatom Definition Flash (Siemens, Munich, Germany) was used to acquire images as a series of transverse sections with a reconstituted voxel size of 0.5 × 0.5 × 5 mm. We conducted a 20 s end-expiratory pause without ventilator disconnection to scan the whole-lung volume. The scans were segmented by 3D Slicer version 4.10.2 (https://www.slicer.org). Segmentation does not include the mediastinum, diaphragm, inferior vena cava, and hilar vessels. Lung CT images were divided into four different functional compartments^31^:

- Over-distended volume: − 1000 to − 901 HU
- Normally aerated volume: − 900 to − 501 HU
- Poorly aerated lung: − 500 to − 101 HU
- Atelectasis or Collapse: − 100 to + 100 HU

An example is presented in Fig. 1D. The mass of each lung fraction (e. g., collapse) was then calculated using the mean density and volume of each fraction assuming that the lung is composed solely of air and water. In previous work^23^, we compared a simple lung heterogeneity index using the ratio between atelectasis and over-distended volume to IST ratio indices. However, since there is a lack of clinical validation for this latter CT metric, we applied Cressoni’s method to quantify the inhomogeneity of the lung^14^.

According to Cressoni’s method, validated in 148 ARDS human subjects, the heterogeneity of a single voxel was measured by calculating based on one voxel’s HU density compared to the density of the voxels surrounding it, from above R_1_ = 2.41 mm and R_2_ = 3.675 mm, Fig. 1C^14^. According to Cressoni, et al., CT-derived lung inhomogeneity (termed H_CT_ in this paper) could be calculated by a ratio of pathologic regions (index showing a value greater than the 95th percentile of the control group—1.61) versus normal regions (index less than 1.61) ^14^.

### Statistical analysis

Statistical analyses were performed in GraphPad Prism 9 (GraphPad Software, La Jolla, CA, USA; https://www.graphpad.com/). Pearson’s correlation highlights the primary results by identifying the correlation between paired repeated measurements of H_IST_, and H_CT_. After that, linear regression analysis was applied to assess the agreement between lung heterogeneity measured with IST (H_IST_) and that measured with CT (H_CT_). For repeated measurements at the same PEEP level, average values were calculated. The changes in lung heterogeneity (ΔH), throughout the different levels of applied PEEP, were reported by the four-quadrant plot^32^. ΔH was calculated by subtracting one absolute H_IST_(or H_CT_) from its value at the preceding PEEP level. The distribution of the data was tested for the normal distribution, as well as, and the mean and standard deviation that were reported.

## Results

We obtained a total of n = 84 paired measurements, including both IST and CT scans at four incremental and decremental PEEP levels. The changes in absolute values and changes in lung heterogeneity were captured and analysed. The changes in V_D_/ELV and V_D_/V_T_ ratios are also reported. The IST heterogeneity measurements had good repeatability, with variations in the same condition being less than 10%.

**Figure 2:**
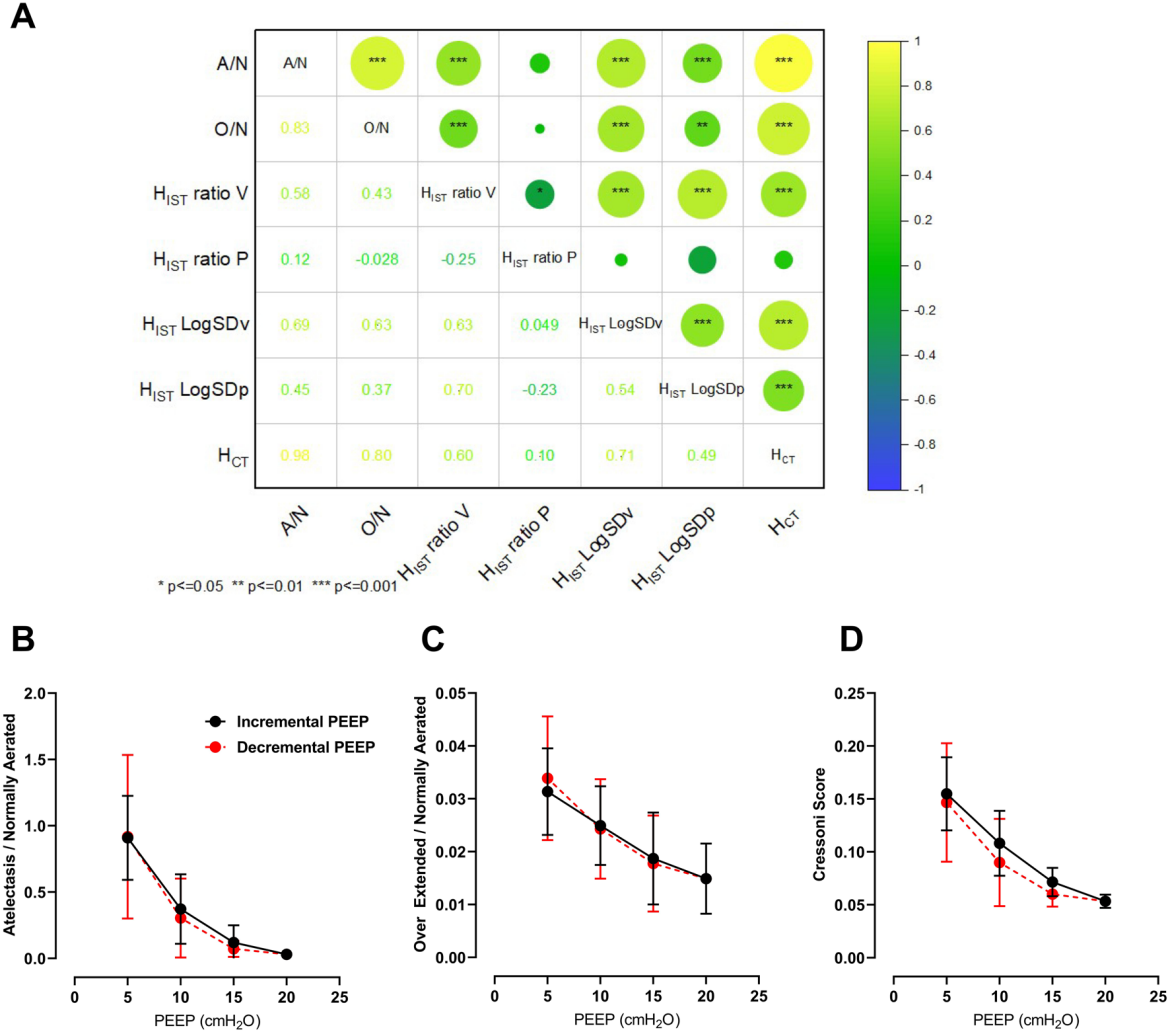
The correlation matrix of the lung heterogeneity was measured by the CT scan versus IST values (panel **A**) and the heterogeneity values vs PEEP changes (panels **B**, **C** and **D**). In panel (**A**), Pearson correlation values are in the lower triangular and the p-values are in the upper triangular. A/N is Atelectasis over Normally aerated volume; O/N is Over-distended over Normally aerated volume; H_IST_ is the IST Heterogeneity including v—ventilation and p—perfusion; H_CT_ is the Cressoni’s Heterogeneity. Panels (**B**) and (**C**) show mean and standard deviation values of the ratio between atelectasis and normally aerated lung against PEEP, and the overextended volume over the normally aeriated ratio against PEEP. Panel D shows the H_CT_ versus PEEP.

Figure 2A shows the Pearson’s correlation and *p* values of heterogeneity results in both CT scanning and IST methods. There was a strong correlation between H_CT_ and both ventilation and perfusion heterogeneity measured by the IST. Ventilation heterogeneity (either measured by the ratio, r = 0.60, *p* < 0.001, or the LogSDv, r = 0.71, *p* < 0.001) had stronger correlations to the H_CT_ than perfusion heterogeneity (measured by the ratio, r = 0.10, *p* > 0.05, or LogSDp, r = 0.49, *p* < 0.001). Interestingly, in the method using LogSD, both ventilation and perfusion heterogeneity correlated with H_CT_ (*p* < 0.001). However, in the ratio method, only the ventilation ratio correlated with the CT (*p* < 0.001).

Furthermore, to validate Cressoni’s score (H_CT_), we also compared its correlation to the aerated and over-extended volumes/normal aerated volumes. H_CT_ correated well with both (A/N, r = 0.98 and O/N, r = 0.80, *p* < 0.001) in Fig. 2A. Figure 2B–D show the overall influence of PEEP on heterogeneity measured by CT images. The difference between incremental and decremental PEEP levels was negligible (continuous lines vs. dash lines in Fig. 2B–D, differences were < 10% of the mean values). When PEEP increased, the lung became more homogeneous. Also, both the ratios of atelectasis: normal aerated lung (2C) and over-extended: normal aerated lung (2D) decreased when PEEP increased. A similar result was seen in H_CT_.

**Figure 3:**
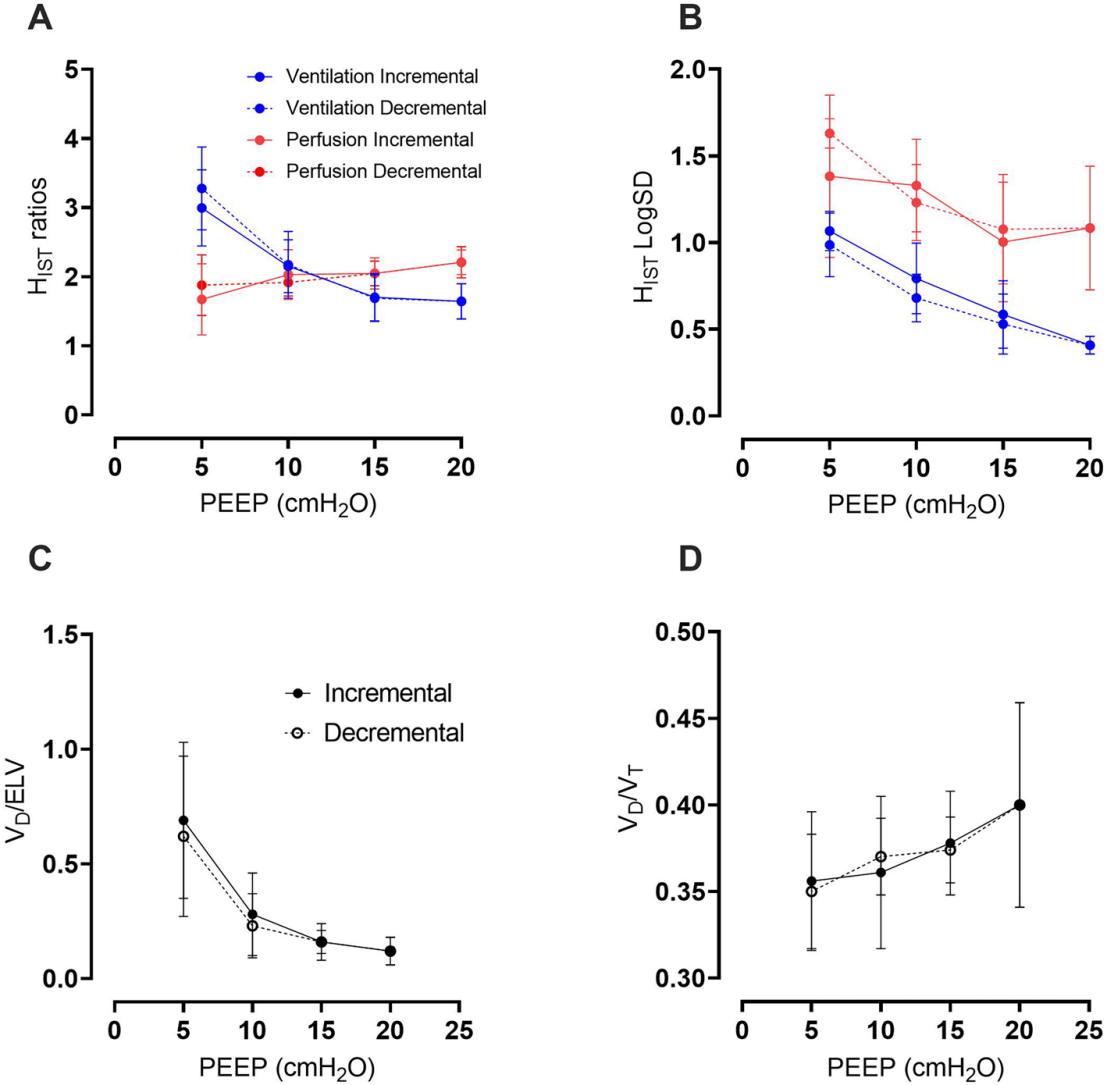
The effect of PEEP on various IST-derived parameters. Panels (**A**) and (**B**) show lung heterogeneity measured by the IST using both ratio and LogSD methods versus PEEP. Solid lines are incremental and dashed lines are decremental PEEP levels. Panels (**C**) and (**D**) show the changes in V_D_/ELV and V_D_/V_T_ ratios versus PEEP. Mean and standard deviation are plotted; black solid lines are incremental and dashed lines are decremental PEEP levels.

Figure 3 shows the impacts of PEEP levels on IST results. Increasing PEEP led to a more homogeneous lung (H_IST_ LogSDv was from 1.0 to 0.4 and H_IST_ LogSDp was from 1.5 to 1.2), in panel b. However, for the H_IST_ratio (in panel a) where the ventilation heterogeneity decreased with increasing PEEP, perfusion heterogeneity worsened. The ventilation and perfusion heterogeneity vs. PEEP plots are seen to cross over at about PEEP 12 cmH_2_O. Furthermore, in Fig. 3C and D, when PEEP levels rose, V_D_/ELV ratios decreased and V_D_/V_T_ ratios increased with negligible variation between incremental and decremental step changes in PEEP.

**Figure 4:**
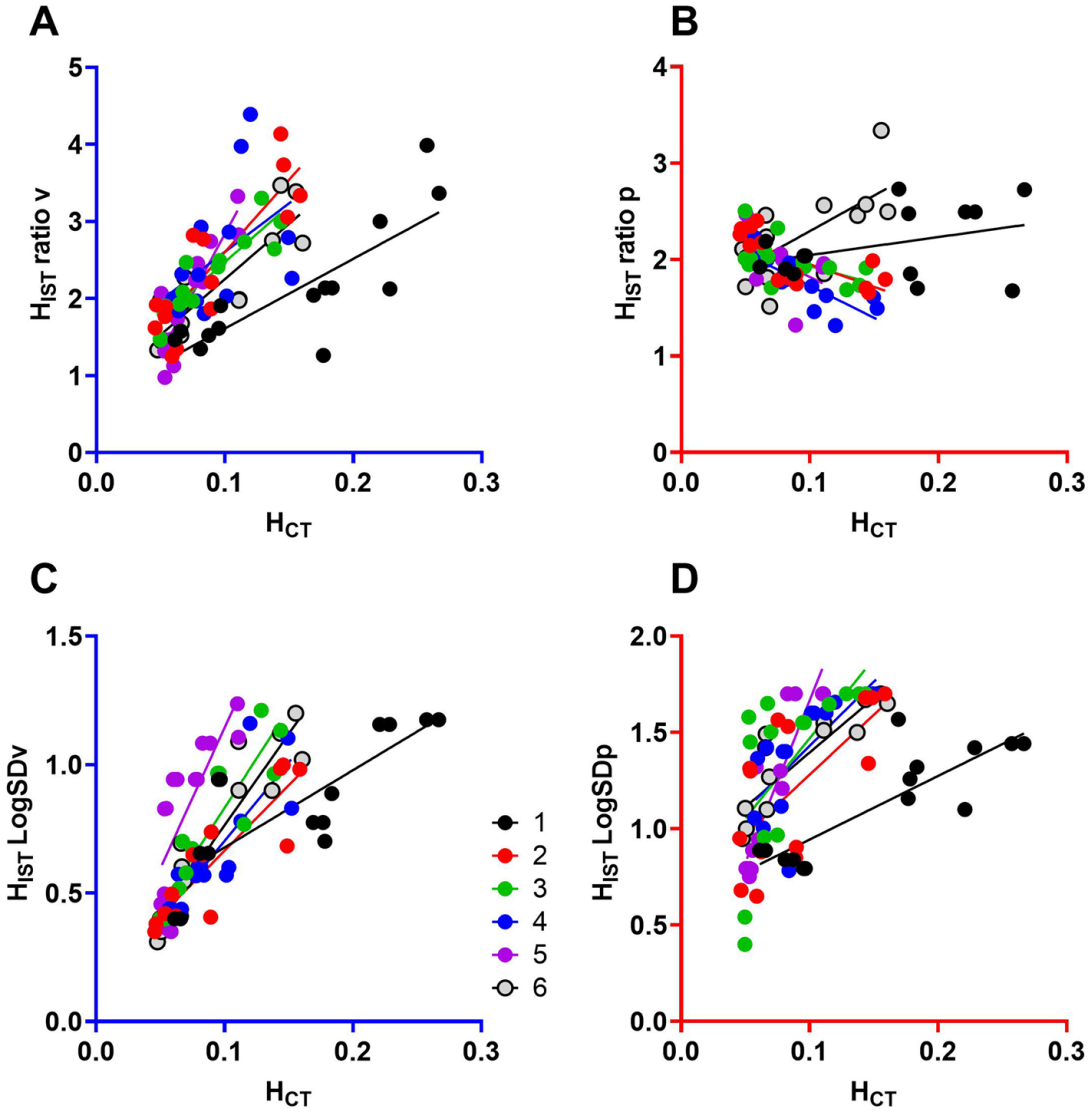
Colour-coded animals in the comparisons of the lung heterogeneity measured by the CT scan and two IST methods. Panels (**A**) and (**B**) illustrate the comparisons of the IST ratios method versus CT. Panels (**C**) and (**D**) illustrate the comparisons of the H_IST_ LogSD method versus CT.

Figure 4 shows the colour-coded scatterplots and linear regression comparison of the lung heterogeneity between IST and CT scanning methods in each animal (numbered 1–6). The full report of the linear regression in individual pigs is presented in Table 2. Due to the contribution of the PEEP level and characteristics of different animals, the correlation of mixed regression results was generally lower than the regression results in individual animals. In individual animals, there was a strong correlation between H_IST_ and H_CT_. For example, the mixed regression of H_IST_ LogSDv versus H_CT_ had R^2^ = 0.50, but in individual animals, the highest recorded, R^2^ = 0.87 was found in animal number 6. Additionally, the H_IST_ LogSD had a stronger correlation with H_CT_ values than the H_IST_ ratios. In the perfusion heterogeneity comparison (Fig. 4B,D), the results of H_IST_ ratios perfusion did not correlate with the H_CT_, while there was only a weak correlation between H_IST_ logSDp and H_CT_.

**Table 2:** Linear regression analysis results in comparison between IST lung heterogeneity indices (H_IST_) and H_CT_ in individual animals with different PEEPs considered a random effect.

Animal number	H_IST_ ratio v		H_IST_ ratio p		H_IST_ LogSDv		H_IST_ LogSDp	
	r	R^2^	r	R^2^	r	R^2^	r	R^2^
1	9.04	0.66	1.88	0.14	2.48	0.51	3.39	0.56
2	18.76	0.74	-4.68	0.54	5.11	0.79	6.22	0.45
3	14.67	0.82	-3.67	0.31	7.62	0.86	8.20	0.39
4	12.60	0.24	-6.67	0.67	6.14	0.69	6.76	0.52
5	30.90	0.80	-6.62	0.26	11.01	0.61	15.88	0.81
6	14.03	0.74	7.39	0.47	6.98	0.87	4.93	0.57
Overall	8.51	0.33	0.66	0.01	3.95	0.50	3.37	0.23

r is for slope, R^2^ is for coefficient of determination, v is for ventilation and p is for perfusion.

**Figure 5:**
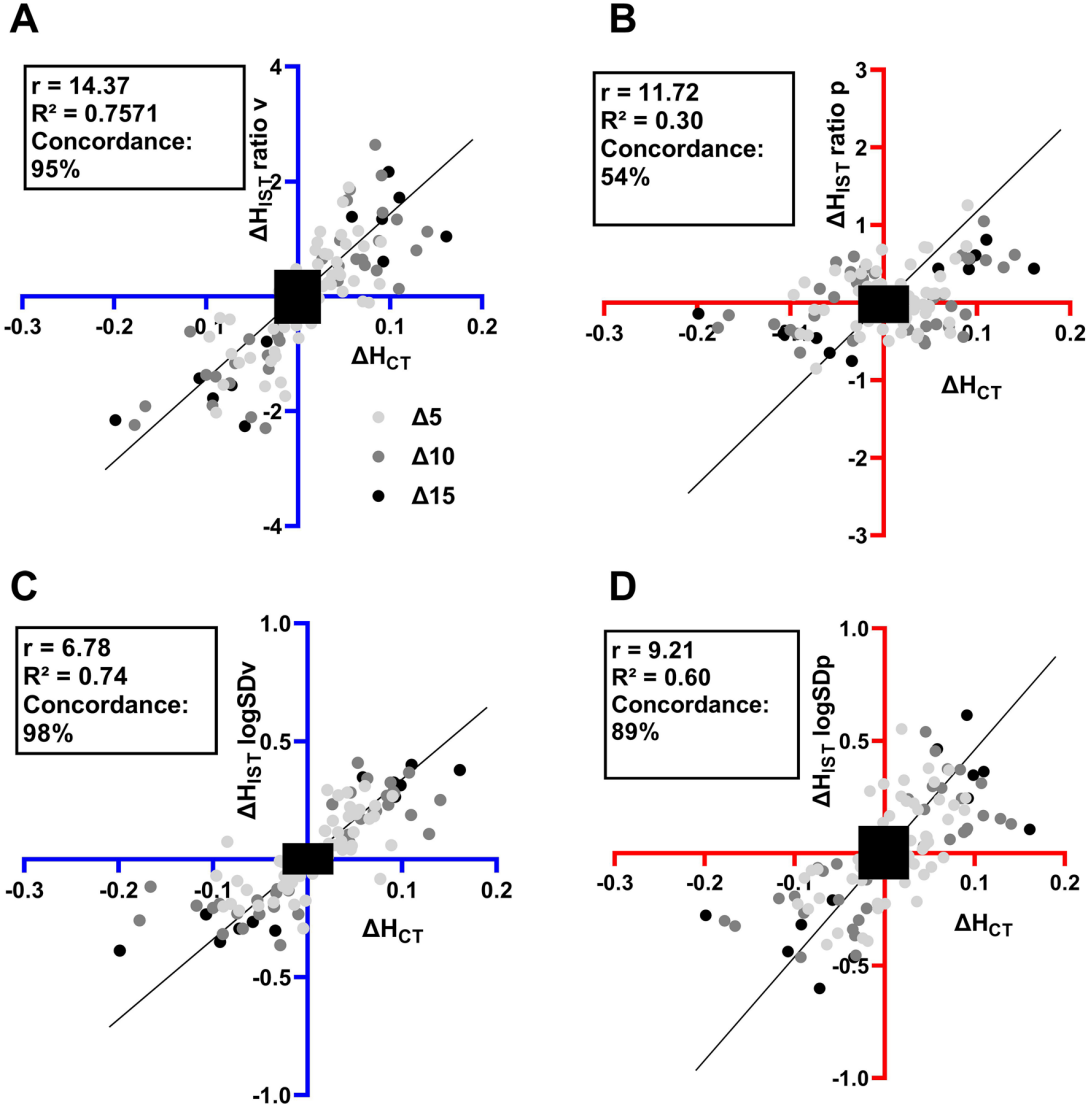
Four quadrant plots show the changes in lung heterogeneity measured by the IST versus CT image with the changes in PEEP level. Panels (**A**) and (**B**) illustrated the comparisons of the H_IST_ ratios method versus H_CT_. Panels (**C**) and (**D**) illustrated the comparisons of the H_IST_ LogSD method versus H_CT_. The exclusion zone (black rectangle) is 15% of the maximum values. R^2^ and slope values are from linear regressions. The concordance rate is the percentage of data points, which are in the $I\left( { + ; + } \right)$ and $III\left( { - ; - } \right)$ quadrants, not including the exclusion zone.

Figure 5 shows the four quadrant plots demonstrating the relationship of the *changes* in heterogeneity in response to a change in PEEP, measured by CT and IST. These comparisons show strong concordances. The $\Delta {\text{H}}_{{{\text{IST}}}} {\text{ LogSD}}$ (panels c and d) showed better concordance with the $\Delta {\text{H}}_{{{\text{CT}}}}$ than $\Delta {\text{H}}_{{{\text{IST}}}}$ ratios (panels a and b). In panels c and d, the concordance of $\Delta {\text{H}}_{{{\text{IST}}}} {\text{ logSD}}$ to the $\Delta {\text{H}}_{{{\text{CT}}}}$ was 98% in ventilation and 89% in perfusion. Additionally, in the perfusion, the concordance of the $\Delta {\text{H}}_{{{\text{IST}}}}$ ratio was 54% (R^2^ = 0.30), which was much lower than 89% (R^2^ = 0.60) of the $\Delta {\text{H}}_{{{\text{IST}}}} {\text{ LogSD}}$.

## Discussion

In this study, we compared lung heterogeneity measured by a bedside-deployable method (Inspired Sinewave Technique) versus CT scan images. Agreements in both absolute heterogeneity values and induced changes in heterogeneity were observed. The negative correlation of PEEP on lung heterogeneity is shown at four different PEEP levels. There was good repeatability of the IST heterogeneity measurement (less than 10% differences in the same condition). Furthermore, when comparing PEEP-induced changes in ventilation heterogeneity, IST indices were > 95% concordant with CT.

There is currently no ‘gold standard’ bedside method to quantify lung heterogeneity. However, researchers point out that the degree of ventilation heterogeneity increases with the severity of lung injury, and it is an important marker of it^24,33^. Normally, the airway wall expanded by the airflow is proportional to the pressure change. However, in the mismatched ventilation region, this caused a high driving pressure significantly to the airway wall, leading to the increasing shear stress between alveolus^34^. In this study, we quantified and compared the absolute changes in heterogeneity using both the IST and CT scan analysis. The CT scan images were processed by Cressoni’s method and used as the ‘gold standard’ for comparison. However, each of these techniques has limitations. Both the acquisition and the analysis of the CT image is labour intensive, and not without clinical risks in transporting critically ill patients to and from the scanning facility. Electrical Impedance Tomography (EIT) shows promise in imaging ventilatory heterogeneity, but it requires a robust quantitative framework to reconstruct data and obtain helpful data^35^. Furthermore, it is possible that the fidelity of EIT in detecting heterogeneity could be improved when combined with IST data. This approach may be a particularly useful fusion because IST alone provides quantification of ventilation distributions only in the statistical sense, whereas EIT provides spatial detail.

The measurement of lung heterogeneity is a newly developed feature of IST. The validation of pulmonary cardiovascular parameters measured by the IST was validated elsewhere^29,30^. In this paper, we validated two IST methods to quantify lung heterogeneity in comparison with CT scans. The ‘IST ratio’ is the ratio of ELV values obtained at tracer periods 180 and 60 s (ELV_180_/ELV_60_) and is a simple representation of the ventilation heterogeneity distribution within the lung. Similarly, Qp_60_/Qp_180_ presents the perfusion heterogeneity distribution. A further method, using lung simulation to characterise the log-normal distribution of ventilation and perfusion, shows advantages compared to the first method. Overall, compared to Cressoni’s score, the logSDv method (r = 0.71, *p* < 0.001) had a stronger correlation than the IST ratio of ventilation (r = 0.60, *p* < 0.001). Both IST heterogeneity measurements have their strengths and weaknesses. The IST ratio is quick to implement in near real-time (less than 10 min per test). However, the IST logSD method is more robust and reliable in comparison to the CT scan but requires more computing resources for the optimisation process (about 10 min in a standard office desktop—core i7, GPU NVIDIA RTX 3050 Ti). Therefore, depending on the purpose of use, both IST methods could be used for bedside monitoring applications or clinical research.

Cressoni’s method considers regional mechanical inhomogeneity on a voxel-by-voxel basis, at the level of the acinus. We have shown that Cressoni’s score correlates well with heterogeneity on a more global scale, as represented by the ratios of atelectatic: normal aeration and over-extended: normal aerated volumes on CT, Fig. 2. We performed the linear regression analysis to compare IST and CT heterogeneity, however, due to the complexity of the PEEP levels and characteristics of individual animals, the results for individual animals had a higher correlation to the mixed regression model (Fig. 4 and summary in Table 2). Although absolute values comparison between IST and CT were not well correlated, *changes* in heterogeneity ($\Delta H$) in response to a change in PEEP, measured by CT and IST have high concordance, 95% and 98% for ventilation and 54% and 89% for perfusion (Fig. 5). Since this global heterogeneity is detected by the IST, this result suggests that the IST heterogeneity is also a marker of mechanical heterogeneity through the lung parenchyma.

There is no agreed universal means of determining the best PEEP, and indeed; perhaps the concept of best PEEP itself is questionable^6,7^. In this paper, we present a means of determining several key lung parameters including lung volume, pulmonary blood flow and the heterogeneity of ventilation and perfusion. Consideration might be given to the concept that PEEP should be applied according to its ability to reduce heterogeneity in ventilation without compromising heterogeneity in perfusion. The study of this trade-off could be the subject of future research.

This research contains several limitations. There is no comparison between healthy versus injured lungs in this research because the experiments were performed in parallel with other experiments, which imposed time and protocol restrictions. The degree of heterogeneity between healthy and injured lungs could potentially be an interesting comparison study. The data collected in this experiment were performed in parallel with another experiment, therefore, the comparison might contain further limitations, such as FiO_2_ levels were not similar. FiO_2_ levels might be an important factor in the distribution of ventilation and pulmonary blood flow inside the lung. However, in this study, there was no significant correlation between FiO_2_ and the reported heterogeneity indices (Supplementary Table A1). We experimented with guidelines to obtain objective outcomes. Furthermore, we used the CT images processed by Cressoni’s method as “gold standard”. However, there are several quantitative methods to interrogate the heterogeneous lung, such as EIT^36^ and hyperpolarized gas MRI^37^. Nevertheless, this work may support useful and more practicable monitoring of ARDS patients under mechanical ventilation. Further research in ventilated patients is needed.

The IST could measure lung heterogeneity noninvasively at the bedside. In this animal study, the heterogeneity of the lung measured by the IST had a strong correlation with the heterogeneity measured with CT scan image analysis. This IST technology and approach open the possibility that PEEP may be optimised for individual patients, aiming at reducing lung heterogeneity. This study supports the implementation of the IST in ventilated patients in future clinical research.

### Supplementary Information

Supplementary Information.

## Data Availability

The datasets used and/or analysed during the current study available from the corresponding author on reasonable request.
