# Supplementary material for: Quantifying heterogeneity in an animal model of acute respiratory distress syndrome, a comparison of inspired sinewave technique to computed tomography
Source: Sci Rep. 2024 Feb 28;14:4897. doi: 10.1038/s41598-024-55144-z (PMC10902369; doi:10.1038/s41598-024-55144-z)
Supplement: Supplementary file 1 — Supplementary Information. [file 41598_2024_55144_MOESM1_ESM.docx]

Supplementary Figure A1: Timeline summary for individual measurement.


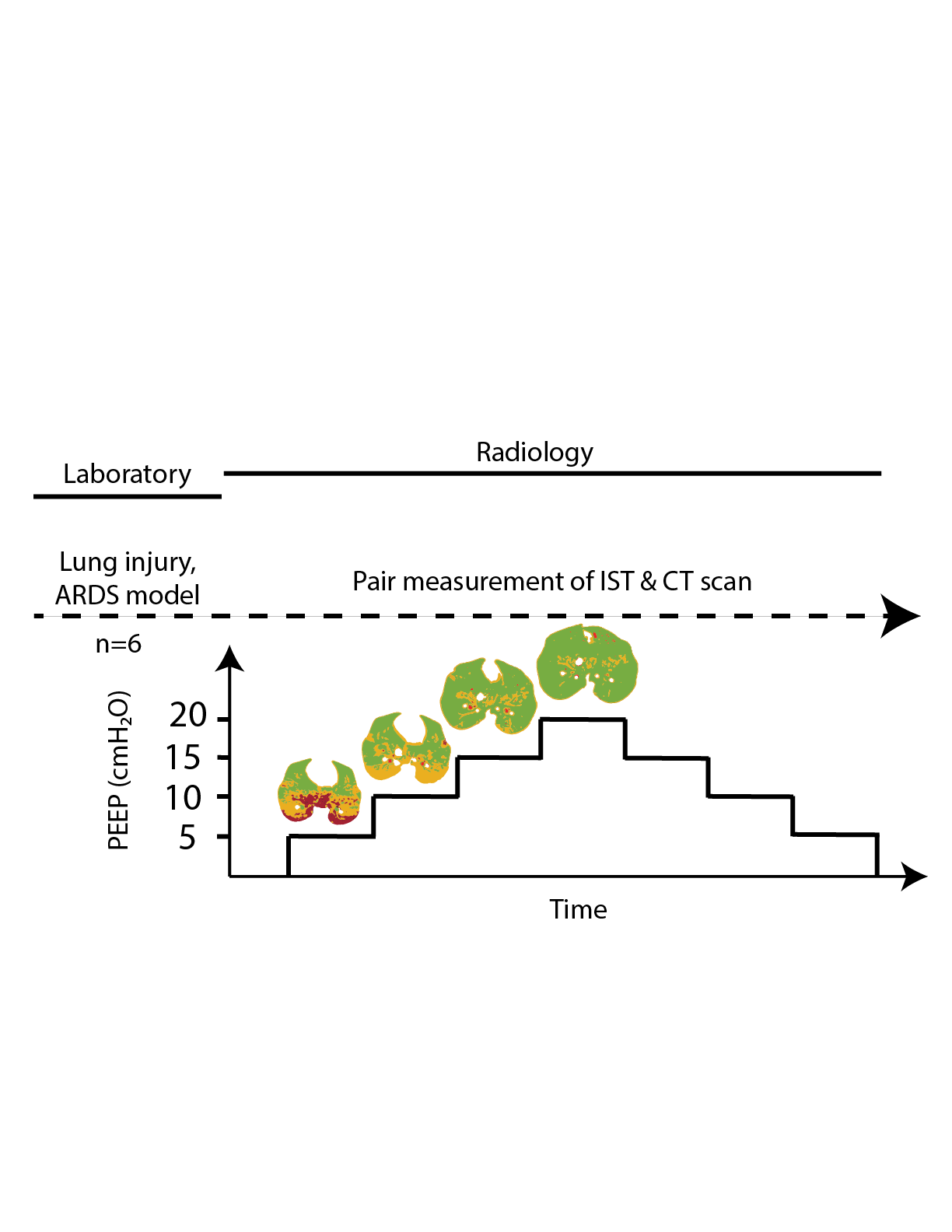


Supplementary Table A1: Summary of Pearson’s correlation between FiO_2_ vs heterogeneity indices measured by the IST in 6 animals at the PEEP level of 5 cmH_2_O.

|  |  | **H_IST_ ratio v** | **H_IST_ ratio p** | **H_IST_ LogSDv** | **H_IST_ LogSDp** |
| --- | --- | --- | --- | --- | --- |
| **FiO_2_ vs Heterogeneity** | r | -0.29 | 0.67 | 0.68 | -0.18 |
|  | p-value | 0.57 | 0.15 | 0.14 | 0.73 |
